# Nurses’ perceptions of the challenges involved in providing of end-of-life care to people with heart failure: a context-based study

**DOI:** 10.1186/s12904-023-01305-2

**Published:** 2023-11-15

**Authors:** Mostafa Akbarian-Rokni, Marjan Mardani-Hamooleh, Mohammad Abbasi, Naima Seyedfatemi, Sally Pezaro

**Affiliations:** 1 Department of Nursing, Nursing and Midwifery Care Research Centre, Iran University of Medical Sciences, Zafar St, Tehran, 1996713883 Iran; 2 Research Centre for Healthcare and Communities’ at Coventry University, Coventry University, Coventry, UK

**Keywords:** Endoflife, Heart Failure, Nursing care, Qualitative research, Palliative care

## Abstract

**Background:**

High quality end-of-life care is essential. Yet for those experiencing heart failure, the provision of high-quality end- of -life care can be challenging. The aim of this study was to explore Iranian nurses’ perceptions of the challenges involved in providing of end-of-life care to people with heart failure.

**Methods:**

Conventional content analysis was used to analyze qualitative data collected from nurses (n = 33) using semi-structured and individual interviews. Participants were aged between 29 and 51 years. The majority of participants were women (n = 20). Most had a bachelor’s degree (n = 24), and work experience of between 7 and 18 years.

**Results:**

Nurses challenges in providing end-of-life care to those with heart failure included (1) adverse consequences relating to compassion fatigue and continued futility in care and (2) lack of palliative care services with regards to a lack of specialists, lack of support from health systems, and poor teamwork.

**Conclusions:**

This is the first qualitative study to explore Iranian nurses’ challenges in providing end-of-life care to those with heart failure. Investment is required in education and research in this area. Particular attention must be paid to prevention of compassion fatigue. Law changes would enable the delivery of higher-quality palliative care in this context overall.

**Supplementary Information:**

The online version contains supplementary material available at 10.1186/s12904-023-01305-2.

## Introduction

Heart failure is characterized by a group of symptoms, including shortness of breath, lower limb edema, fatigue, anxiety, and pulmonary congestion leading to reduced cardiac output, where the heart can no longer pump blood adequately [1]. Heart failure is considered a major global public health issue [2], and is the most common reason for hospitalization of people over 65 years old. People with heart failure experience re-hospitalizations, which impose economic and social burdens upon health systems [1]. In addition, frequent hospitalizations have a negative effect upon one’s quality of life [2]. Those with heart failure experience a survival rate of approximately 10% after 10 years [3], though rates of mortality differ worldwide. For example, in the United Kingdom (UK), from 2000 to 2017, heart failure was responsible for the death of 15,048 women and 15,822 men [4]. Yet in Iran, non-adherence to treatment among those with heart failure has created problems such as frequent hospitalizations, poorer health outcomes and a higher mortality rate of 18.2% [5].

End-of-life care is provided to patients with heart failure during the last days and weeks of life. In clinical practice, we observe that when these patients have an ejection fraction below 10% and become dependent on dopamine, they are often considered to be at the end of their life and thus need to receive end-of-life care. End- of- life care may also be required where people with heart failure are at risk of death. The end-of-life care provided by nurse is of particular importance as they can focus on facilitating patient choice, and managing symptoms such as pain, without increasing the risk of death [6]. In such cases, end-of-life discussions with nurses are crucial [7], particularly as those with heart failure often experience sudden and unexpected changes and deteriorations in their condition [8]. Nevertheless, it is often unclear how to best approach such conversations from the perspective of clinicians.

A recent review of the literature highlighted the importance of analyzing the views of care professionals regarding the provision of end-of-life care for people with heart failure in order to understand how their care performance is affected [9]. Elsewhere, health professionals have identified the lack of an accurate prognosis of advanced heart failure a barrier to providing quality end-of-life care discussions [10]. Family caregivers who provide care at home for people with heart failure also need to engage in high quality conversations about end- of- life care [11]. Yet while the literature has explored challenges relating to end-of-life care discussions for those with heart failure from the perspective of doctors and family caregivers, the nurses’ point of view in this field has received less attention. Families’ perspectives and nurses’ perceptions of the challenges in providing end-of-life care and related discussions have not yet been analyzed in Iran at all. This is concerning given that nurses provide the majority of end-of-life care and more often initiate associated conversations, both of which can improve quality of life of patients with heart failure. Considering the above, the aim of this research was to explore nurses’ perceptions of the challenges involved in providing of end-of-life care to people with heart failure.

## Methods

### Design

This qualitative study invited Iranian nurses to offer their perceptions in relation to the challenges in providing of end-of-life care to people with heart failure using semi-structured and individual interviews. Conventional content analysis was used to make sense of the data collected [12].

### Participants

The research environment included two cardiac referral educational centers in Tehran, the capital of Iran, where those with heart failure patients are treated and cared for. Purposeful sampling was used to recruit participants, whereby nurses in full-time employment who had provided care to those with heart failure patients at the end- of- life (> six months) were invited to participate. Sampling was done with maximum variation in terms of age, gender, education level, work experience, and experience of providing end-of-life care to those with heart failure.

### Data collection

After receiving information with regards to the study, the head nurses were asked to introduce eligible qualified nurses to the research team. We developed a guide for the interviews according to the aim of this study (Appendix 1). The interview guide questions were designed based on the experiences of the author, who is a qualitative researcher and has experience of end-of-life care for patients with heart failure. All participants provided their consent in writing after being informed about the study aim. Subsequently, semi-structured interviews were conducted and face to face to collect data. Each interview lasted between 35 and 50 min. The interviews were conducted between January and March 2022. A total of 33 individual interviews were conducted, each at a time most convenient to the participants, whilst not on duty in a private conference room on hospital premises. Data collection and recruitment ended once the data were considered well saturated as the trustworthiness of content analysis depends upon well-saturated data [13].

### Data analysis

Data collection and analysis were done simultaneously. In order to analyze the data in line with qualitative content analysis [12], each interview was recorded and typed verbatim. Subsequently, after reviewing the interview text, semantic units were extracted from them. Afterwards, codes were assigned to similar semantic units. Later, all codes were placed in subcategories based on their similarities and differences. Finally, all subcategories were grouped under overall main categories.

### Trustworthiness

Credibility, dependability, confirmability and transferability criteria were used to determine rigor [14]. In order to determine credibility, continuous engagement was maintained with both the subjects and the data. The reflections of the research team were also used in connection with the process of data collection and analysis. Findings were discussed with a subset of participants as well as two experienced researchers in nursing. According to Lincoln and Guba (1985), dependability ‘seeks means for taking into account both factors of instability and factors of phenomenal or design induced changes’, that is, the degree to which data change over time and alterations made in the researcher’s decisions during the data analysis process [14]. In order to determine dependability, an external observer who was familiar with both the clinical environment and qualitative research, but was not a member of the research group, was asked to examine the process of data analysis and the subsequent findings. Confirmability refers to “the extent to which the characteristics of the data, as posited by the researcher, can be confirmed by others who read or review the research results” [14]. To determine confirmability, all the activities were recorded, and a report of the research process was prepared. In order to determine transferability, findings were discussed and confirmed with two nurses external to the study with a history of relevant end-of-life care.

## Results

A total of 33 nurses aged between 29 and 51 years participated. The majority of participants were cisgender women (n = 20). Most had a bachelor’s degree (n = 24), whereas all others had a master’s degree. Participants had a work experience of between 7 and 18 years. Their history of caring for end-of-life those with heart failure varied from 6 months to 11 years.

Overall, the challenges to nurses providing end-of-life care to those with heart failure were determined and classified in two main categories (1) adverse consequences of end-of-life care and (2) lack of palliative care services. Subcategories relating to adverse consequences of end-of-life care included (1) dealing with compassion fatigue and (2) continued futility in care. Subcategories relating to lack of palliative care services included (1) lack of specialists, (2) lack of support from health systems, and (3) poor teamwork. These main categories and subcategories are described below, with salient quotes used to highlight some of the particular meanings identified.

### Adverse consequences of end-of-life care

According to nurses, there is a diversity in adverse consequences when providing end-of-life care to those with heart failure. On the one hand there is nurses dealing with compassion fatigue. On the other hand, there is seemingly a continued futility in providing care. This dichotomy seemingly ignited emotional conflict and a degree of distress among participants, whose professional identity grounded them toward a desire to do the best for their patients, despite the adverse consequences.

#### Dealing with compassion fatigue

Providing end-of-life care to those with heart failure was perceived to affect the mental wellbeing of nurses. Indeed, the narratives of nurses indicated that they had been experiencing compassion fatigue due to prolonged immersion in the grief and suffering of those affected by heart failure. Beyond compassion fatigue, participants describe nightmares, associated physical symptoms and reference feelings of anxiety and worthlessness. This is concerning as the poorer physical, emotional, social and spiritual health of healthcare providers associated with compassion fatigue can impact the delivery of services [15].

*“Keep taking care of end-of-life heart failure patients has made me nervous. I am constantly anxious. I feel very tired and have headaches and dizziness, and these are really painful because I think I am drowning in the suffering of these patients. I must say that under these challenging conditions, the quality of care for those patients who really have nothing left at the end of their lives is greatly reduced.“* (Participant (P) 5).

*“Seeing the death of heart failure patients affects me emotionally and makes me feel worthless. I think so much about the patients who are on the verge of death and then die. It even affects my relationships with others so that I distance myself from friends, acquaintances, and relatives… You may not believe it, but I see nightmares of the dead patients at night because dead patients come to my sleep.“* (P11).

#### Continued futility in care

When there is no opportunity to prolong the life of patients with heart failure, from the perspective of nurses, there is seemingly little point in continuing to provide care for them. In this sense there was seemingly lower job satisfaction where nurses were no longer able to preserve life, particularly where there was little instruction as to what to do in such scenarios. This perceived futility may be indicative of the role of the nurse being in conflict with the demands of the service in this context. For example, the following participant describes the prolonging of life being futile where death is inevitable. Such reflections may suggest that end-of-life care is not designed for dignity in this context, which professionally conflicts with the role of the nurse.

*“We don’t have a single instruction in the country that allows us to take care of a heart failure patient at the end- of- life in such a way that futile care is not repeated for the dying patient! For example, why should care be continued for patients who have an ejection fraction of less than 10% for a long time and there is no hope for their progress?”* (P24).

Indeed, as the following participant describes, it is law which promotes the prolonging of life in favor of dignity in dying. Such conflicts with the professional role of the nurse may cause further emotional harm for nursing professionals.

*“When a patient becomes dependent on dopamine, if dopamine is cut off, he will surely die. Such patients have no resuscitation order and caring for them is practically a futile task… However, the same patient should be treated and receive CPR, if necessary, because in our country, no resuscitation law has not yet been approved by the Ministry of Health!“* (P8).

### Lack of palliative care services

Nurses’ narratives indicated that service-related factors such as a severe shortage of specialists, a lack of support from health systems, and poor teamwork led to challenges in providing adequate palliative care. In this context, palliative care is used to describe a broad concept inclusive of end-of-life care.

#### Severe shortage of specialists

The lack of specialist medical professionals in the field of palliative care is challenging when providing end-of-life care for those with heart failure. With such a lack of services, some illnesses and geographical areas were perceived to be prioritised for end-of-life care above others. This was frustrating to participants, who equated this to being notably underserved and under recognised in their area of work.

*“While palliative care can improve the quality of life of heart failure patients, in our country it is practically ignored for heart failure patients because we do not have palliative care specialists. In Iran, palliative care is only for cancer patients, and even it does not exist for everyone or all parts of the country. Now, although we are in the capital, we don’t even have a doctor specializing in palliative care for heart failure patients”.* (P19)

Nurses reported that in particular, the lack of a master’s degree program in palliative care in our universities leads to a lack of palliative care nursing specialists to provide end-of-life care for these patients. This, partnered with a lack of priority setting in this area was perceived to be a key barrier in providing high-quality end-of-life care. Such findings indicate a need for enhanced education, and recognition of the need for specialized palliative care nurses in this speciality.

*“In Iran, there is no master’s degree in palliative care at universities, as a result, specialist nurses are not trained in this regard. Consequently, the lack of palliative care specialists does not allow palliative care at the end-of-life to be implemented for patients in general and for heart failure patients in particular.“* (P30).

#### Lack of support from health system

One of the challenges of end-of life -care perceived by nurses was the lack of support from health system to provide palliative care services for those with heart failure patients at the end- of their life. This, partnered with a lack of specialists in the field, led to further sentiments conveying a lack of respect and value placed upon high quality end-of-life care in this context. One suggestion for this was driven by perceived financial incentives.

*“The main problem is that palliative care for heart failure patients has no place in Iran… Palliative care services do not generate income for the government, so it is not worth it for the government to set up palliative care centers for patients. In fact, the health system does not provide any support in this regard. This causes us to face a severe lack of palliative care services for people with heart failure, especially at the end-of-life phase.“* (P15).

Nurses understood the value they could potentially bring to those with heart failure in need of high-quality end-of-life care. Despite this, they remained frustrated that the benefits they could bring had not been considered or prioritised, as the following quote demonstrates;

*“Palliative care can meet the physical, spiritual and psychological needs of heart failure patients at the end- of- life, but the problem is that centers for this type of care have not been considered by the health system, and I must say that health system has no support approach in this context.“* (P21).

#### Poor teamwork

High quality teamwork was perceived to be important in the provision of high-quality end- of- life care. However, teamwork was perceived to be poor and fragmented, leading to an absence of palliative care services when required. Communication, a key competence of nursing professionals, was considered to be key in this, particularly with regard to interprofessional working. Yet seemingly no training or processes were in place to facilitate this. Evidently, distinct “*attention should be paid to the communication between people from different professions in the form of a palliative care team”* (P27).

*“In my opinion, teams that provide palliative care to heart failure patients are weak and fragmented in terms of communication. In order to provide palliative care to these patients, communication between different specialists in interdisciplinary teams must be coherent and coordinated and effective interactions should exist… For example, for spiritual counseling, the relevant specialist should cooperate with the cardiologist. Also, the psychologist and nurse should cooperate with the rest of treatment team, but the fact is that the team communication is very weak, and this causes a big challenge for palliative care.“* (P1).

Moreover, nurses described professional conflict with regards to medical and nursing staff as being something which would further hinder progress, even if effective services were to be established. Such professional conflicts may result from gender related oppression with regards to these professions and potential hierarchy in teams. Distinct programs of work to address these conflicts will be key to unlocking the potential of high-quality end-of-life care delivered in this context.

*“I think that even if palliative care was established in our country like the developed countries, the provision of palliative care for heart failure patients at the end-of-life phase still fails, because the medical and nursing staff do not have the team spirit for effective teamwork.“* (P33).

## Discussion

In this study, the challenges of providing end-of-life care for those with heart failure from the perspective of Iranian nurses broadly involved adverse consequences and a lack of palliative care services. In this context, we considered palliative care to be a broader term that covers end-of-life care. Adverse consequences of end-of-life care delivered to those with heart failure included dealing with compassion fatigue and the perceived continued futility in care. This finding is not surprising given that the highest level of compassion fatigue experienced among nurses in the world is related to those working in Asia [16]. By immersing in the sorrow and pain of patients, the nurses experience a decline in the quality of care and a series of disturbing psychological symptoms. Ultimately, the constant exposure of nurses to the pain and suffering of patients exposes them to the risk of compassion fatigue, which endangers both their personal and professional life [17]. Nurses working in China have also been found to experience compassion fatigue while providing care to terminally ill patients [18]. Equally in Turkey, nurses who provided continuous care to chronically ill patients faced compassion fatigue and ultimately, low job satisfaction in this regard [19]. Compassion fatigue has been described as a “dead nightmare”, and many nurses experience compassion fatigue while providing services to patients with life-threatening conditions in their end- of- life care [20]. As such, it will be important to provide support and care to the nursing staff involved, as well as to the patients and their families in improving future services.

Participants referred to the prolonging of life during the end-of-life phase as “futile care”. Currently, in Iranian hospitals, there is no specific protocol that prevents those with heart failure from receiving such ‘futile care’ at the end of their life. Equally, the Ministry of Health has not yet approved the “no resuscitation” law, and nurses must perform mandatory resuscitation on all patients, which is a step towards continued futility in care [10]. In other studies conducted in Iran, nurses reported resuscitation futility in caring for patients at the end- of- life [21, 22]. The provision of futile care when nurses are involved in the direct care of patients at the end-of-life is associated with creating moral problems for nurses at the bedside, making their role in end-of-life decisions for patients less than transparent or appropriate [23]. However, the laws approved by the Ministry of Health of Iran order that no person may end another person’s life. Thus, nurses must continue in this ‘futile’ manner, which was considered to be inappropriate in this context. This is especially true for dopamine-dependent patients and those with a low ejection fraction. As such, laws and regulations in this regard require change.

The lack of palliative care services was perceived to be another challenge for nurses providing end-of-life care for those with heart failure. This manifested in a lack of specialists, a lack of support from health systems, and poor teamwork. Meanwhile, the palliative care needs of those with heart failure are increasing [24]. Our findings demonstrate that Iran’s health system suffers severely from a lack of palliative care services for those with heart failure at the end of their life. The nurses in this study perceived that one of the reasons for this is the lack of palliative care nursing specialists. This obstacle to providing high-quality care has similarly been reported elsewhere [25]. Reportedly in Iran, there is no specialized master’s degree program in palliative care, which would explain the lack of palliative care specialists available to provide palliative care. Thus, our findings emphasize the need to design a palliative care master’s degree curriculum for nurses in Iran.

The lack of support from health systems for the establishment of palliative services was another challenge of providing end-of-life care. In this regard participants perceived that the reason for the lack of support from health systems is that palliative care is not profitable for the government. Yet it is highly important for Iran to have high-quality palliative care services provided by the health system. For example, providing high-quality palliative care to those with heart failure helps to meet their physical, emotional, social, and spiritual needs and improves their quality of life [3]. Such high-quality palliative care can also result in a reduction in referrals to cardiologists, a reduction in CCU/ICU admission, and shorter hospital stays [26]. It also helps to maintain dignity at the time of death [27]. However, it is difficult for those with heart failure to access such palliative care at the end-of-life [9]. Consequently, future research could usefully explore how such services may be improved and better accessed by those who need it most.

Our findings highlight poor teamwork in Iran, which has led to ineffective communication between health care providers in the palliative care setting. The need for cooperation with multidisciplinary team members in providing palliative care for those with heart failure has also been highlighted elsewhere [28]. Thus, team building activities and awareness raising of the importance of team work is required. Moreover, future research in this area may work to strengthen interprofessional relationships.

As we solely solicited the perception of nurses’ challenges in providing end-of-life care for those with heart failure, we suggest future studies may benefit from the perceptions of cardiologists in order to understand these team based challenges more widely. The findings presented here may be useful in providing evidence for an action research study in future. Whilst the purpose of qualitative studies is not to generalize the findings, qualitative studies are dependent on the context in which the phenomenon under study is analyzed [12]. Thus, further qualitative research is required in other contexts to strengthen the body of evidence in this area overall.

## Conclusions

This is the first qualitative study to explore Iranian nurses’ challenges in providing end-of-life care to those with heart failure. The prevention of adverse consequences such as compassion fatigue in healthcare staff must be addressed. Equally, Iran’s Ministry of Health need to formulate clear regulations and design necessary protocols to solve the challenges in providing end-of-life care for those with heart failure. This may go some way to avoiding such palliative care being perceived as ‘futile’. More specialist physicians and nurses in the field of palliative care are required. Higher education courses may assist in this pursuit. Equally, effective teamwork between multidisciplinary team members may be strengthened via future training and research.

### Electronic supplementary material

Below is the link to the electronic supplementary material.

Supplementary Material 1: Additional file 1: Appendix 1: Interview Guide

## Data Availability

The datasets generated and analysed during the current study are not publicly available due to the confidentiality and the traceability of the qualitative data but are available from the corresponding author on reasonable request.
