# Supplementary material for: Nurses’ perceptions of the challenges involved in providing of end-of-life care to people with heart failure: a context-based study
Source: BMC Palliat Care. 2023 Nov 15;22:180. doi: 10.1186/s12904-023-01305-2 (PMC10648333; doi:10.1186/s12904-023-01305-2)
Supplement: Supplementary file 1 — Supplementary Material 1: Additional file 1: Appendix 1: Interview Guide [file 12904_2023_1305_MOESM1_ESM.docx]

**Additional file 1: Appendix 1. Interview Guide**

Main questions:

How would you describe end-of-life care of patients with heart failure?

How would you describe the current situation within your organisation with respect to timely recognition of end-of-life care needs in patients with heart failure?

How would you describe your experiences for timely recognition of end-of-life care needs in patients with heart failure?

What would you need for directing the end-of-life care needs in patients with heart failure?

What makes it difficult to recognize end-of-life care needs in patients with heart failure?

What are the problems of providing this type of care?

Probing questions

What do you mean by...?

Can you give an example in this regard?"

Can you tell me your experience about this?
